# Faecal calprotectin determination: impact of preanalytical sample treatment and stool consistency on within- and between-method variability

**DOI:** 10.11613/BM.2019.010707

**Published:** 2019-02-15

**Authors:** Gordana Juricic, Tina Brencic, Andrea Tesija-Kuna, Milena Njegovan, Lorena Honovic

**Affiliations:** 1 Department of Laboratory Diagnostics, General Hospital Pula, Pula, Croatia; 2 Department of Clinical Chemistry, Sestre milosrdnice University Hospital Center, Zagreb, Croatia

**Keywords:** calprotectin, preanalytical phase, inflammatory bowel disease, immunoassay

## Abstract

**Introduction:**

We assessed the differences in faecal calprotectin (FC) concentrations measured by two assays depending on the stool consistency and extraction method.

**Materials and methods:**

Stool samples were extracted using the EliA Stool Extraction Kit, Calex® Cap extraction device and respective weighing methods, while FC concentrations were measured using the EliA^TM^ Calprotectin and Bühlmann fCAL® Turbo method and checked for within- and between-method variability with regard to extraction method and stool consistency category. Extraction yield was evaluated for impact of different sample incubation time (10 min and 1 h) in extraction buffer for both methods and for impact of different initial sample dilutions (1:50, 1:100, 1:500) for fCAL® Turbo method.

**Results:**

Results determined from Calex® Cap extracts were higher compared to weighing method extracts (mean bias 33.3%; P < 0.001), while no significant difference was found between results obtained with EliA Stool Extraction Kit and weighing method (mean bias 0.1%; P = 0.484), in both cases irrespective of stool consistency. Bühlmann fCAL® Turbo results were higher than EliA^TM^ Calprotectin results (mean bias 32.3%, P = 0.025 weighing method; and mean bias 53.9%, P < 0.001 extraction devices), the difference is dependent on stool consistency and FC concentration. Significantly higher FC extraction yield was obtained with longer sample incubation time for both methods (P = 0.019 EliA^TM^ Calprotectin; P < 0.001 fCAL® Turbo) and with increasing initial sample dilution for fCAL® Turbo method (P < 0.001).

**Conclusion:**

Preanalytical stool sample handling proved to be a crucial factor contributing to within- and between-FC assay variability. Standardization is urgently needed in order to assure comparable and reliable FC results.

## Introduction

Faecal calprotectin (FC) is a stable, feasible biomarker, which is released in stool through neutrophils´ disruption during inflammation in bowel mucosa. Its determination substantially reduces the need for invasive endoscopy with biopsy which is expensive but nevertheless regarded as gold standard for assessing mucosal inflammation (*1*, *2*). As the most widely used faecal biomarker, FC has been recommended for diagnosis and differentiation between inflammatory bowel disease (IBD) and irritable bowel syndrome in adults and paediatric population, for monitoring treatment response, mucosal healing process or predicting disease relapse (*3*-*6*). The lack of overall process standardization, from faecal sample preparation to methodology used for FC measurement, results in great variability between commercially available assays (*7*-*11*). Faecal sample weighing, as the gold standard FC extraction method, is rather impractical and time-consuming; thus extraction devices have been introduced as convenient alternative (*12*). The first version of such device, Smart-Prep faecal sample preparation kit (Roche diagnostics, Manheim, Germany), might be used for all methods, has a sample chamber carrying approximately 85 mg of stool sample and 4 mL of method specific extraction buffer is added to obtain 1:50 dilution (*13*, *14*). Recently, a more elegant variation of extraction device prefilled with method-specific extraction buffer and sampling pin has been introduced. The sample pin grooves are presumed to carry an approximate amount of stool, which is diluted in corresponding buffer volume to obtain method-required dilution (*13*-*16*). Interestingly, it has been noticed that there is an increased FC extraction yield with higher sample dilutions but it has not been properly investigated yet (*17*). Although few authors have performed verification of extraction devices and found them suitable even for patient conducted extractions, findings are still rather indefinite due to either the lack of comparison with the reference weighing method, limited number of study participants, or lack of performance with stool samples of different consistency (*18*-*20*).

In this study we aimed to assess the differences in FC concentrations measured by our routine Bühlmann fCAL® Turbo (Bühlmann Laboratories AG, Schönenbuch, Switzerland) method and EliA^TM^ Calprotectin (Thermo Fisher, Uppsala, Sweden) method, depending on stool consistency and different extraction methods. Furthermore, we wanted to evaluate FC extraction yield with regard to different soaking time and different initial sample dilution in extraction buffer.

## Materials and methods

### Study design

The study was conducted at the Department of laboratory diagnostics, General Hospital Pula (Pula, Croatia) from June to December 2017. The study was approved by the Institution’s Ethics Committee and conducted according to the principles of the Helsinki Declaration. Study included only the leftovers of stool samples consecutively submitted to our laboratory for routine FC determination, thus no additional samples were requested from patients. As our routine practice is to reject stool samples that are older than 3 days, such samples were also excluded from the study. For study purposes only, we excluded samples for which we estimated that the amount of stool provided was too small for multiple extraction procedures. Upon submission, samples were immediately frozen and stored at – 20 ºC for maximum 10 days.

**Figure 1:**
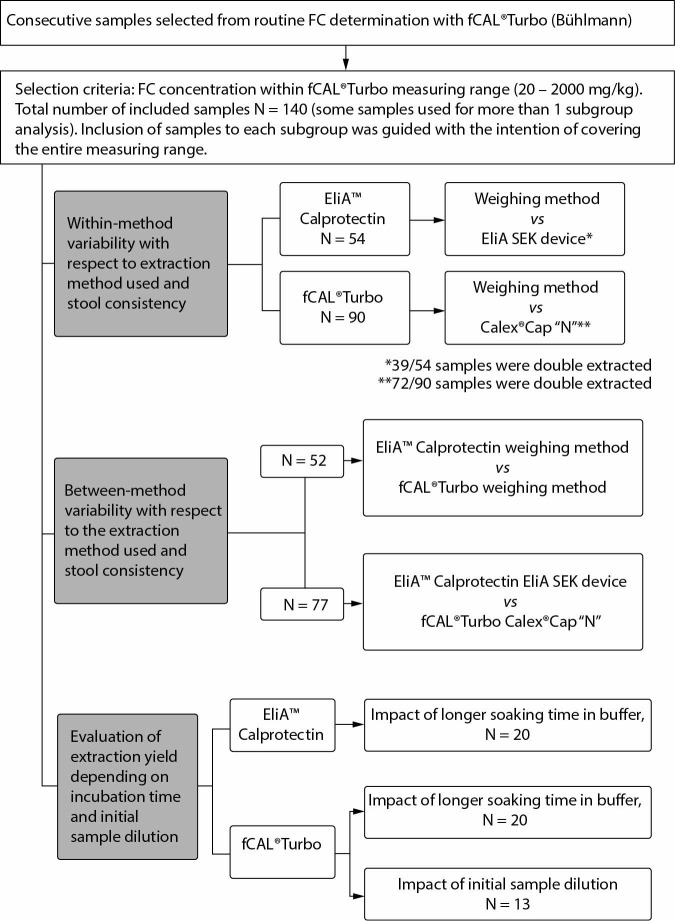
Schematic presentation of study protocol. FC - faecal calprotectin. EliA SEK - EliA Stool Extraction Device.

At the day of routine analysis, samples were left at room temperature for several hours. Samples of normal and liquid consistency were thoroughly homogenized with spatula before extraction. Since hard stool samples are impossible to homogenize, the sample for extraction was picked from different parts of stool samples as instructed by the manufacturer (*16*). Consistency of each stool sample was estimated according to Bristol stool scale (BSS) and categorized in 3 classes: 1) hard (BSS 1 and 2), 2) normal (BSS 3, 4 and 5), and 3) liquid (BSS 6 and 7) (*21*). All stool samples were routinely extracted with Calex® Cap “N” (Calex, Bühlmann Laboratories AG, Schönenbuch, Switzerland) stool extraction device and FC concentration measured with particle enhanced turbidimetric immunoassay (PETIA), Bühlmann fCAL® Turbo method on Roche Cobas c501 analyser (Roche diagnostics, Manheim, Germany). Selection of samples for further study was based on these FC results; *i.e.* samples falling out of the Bühlmann fCAL® Turbo measuring range (20 - 2000 mg/kg) were excluded while taking care of covering the entire measuring range. The study finally included a total of 140 samples, which were divided into 3 subgroups, with some of the samples used for more than one subgroup in study protocol. Final number of samples included in the subgroups and study protocol are summarized in Figure 1.

Immediately after the routine extraction of 140 samples with Calex extraction device and FC determination with fCAL® Turbo, additional extraction methods were performed followed with FC measurement according to predefined subgroup analysis (Figure 1). Extraction with Calex device were performed according to manufacturers’ instructions with the yield of 1:500 ready-to-use diluted extracts (*16*). Additionally, extractions were performed using the EliA Stool Extraction Kit (EliA SEK, Thermo Fisher, Uppsala, Sweden) extraction device on totally 105 samples according to the manufacturers’ instructions to yield 1:50 ready-to-use diluted extracts (*14*). Furthermore, for weighing method extraction, Smart-Prep kit was used. Based on sample weight, a corresponding volume of Bühlmann extraction buffer (totally 117 samples) or EliA extraction buffer (totally 58 samples) was added to obtain basic 1:50 dilution (*13*, *14*).

As mentioned, some of the samples overlapped between different subgroups and were extracted with all four extraction methods described, while some of them were used only for one subgroup and extracted with one or two extraction methods. We were guided with the intention of covering a wide FC concentration range in each subgroup and this was the main criteria for further distribution into the subgroups for analysis.

Independent of the extraction method used, all extracts were thoroughly vortexed for complete dissolution, incubated at room temperature for 10 minutes, and centrifuged for 10 minutes at 3000xg, as recommended by manufacturers (*14*, *16*). For fCAL® Turbo method, supernatants gained with weighing method were additionally diluted 1:10 with buffer to yield 1:500 dilution. All extracts were transferred into empty tubes and analysed immediately after. One experienced laboratory expert performed all extractions. The obtained extracts were used in the study protocol as described below.

### Within-method variability with respect to extraction method used and stool consistency

To assess the difference within Bühlmann fCAL® Turbo method FC results obtained using Calex extracts and reference weighing method extracts, samples additionally extracted with weighing method using the Bühlmann extraction buffer were used. Furthermore, to assess the difference within EliA^TM^ Calprotectin method FC results obtained with extraction device and weighing method extracts, samples extracted with EliA SEK extraction device and weighing method using the EliA extraction buffer were used. Since the FC concentration is related to sample weight and extraction devices, to perform this step, we double extracted some samples (Figure 1) with both extraction devices to gain intra-extraction precision (mean precision of multiple performed extractions with same extraction device).

### Between-method variability with respect to the extraction method used and stool consistency

Extracts obtained with weighing method using method-corresponding buffer were measured with fCAL® Turbo and EliA^TM^ Calprotectin methods, respectively. Furthermore, extracts obtained with corresponding extraction devices were measured with fCAL® Turbo and EliA^TM^ Calprotectin FC methods, respectively.

### Evaluation of extraction yield depending on incubation time and initial sample dilution

To study the impact of longer soaking time in buffer (required for hard stool samples to achieve complete dissolution) on FC results, 20 samples that were homogenized and incubated according to manufacturers’ instructions as described in the previous section, were also left to incubate for totally 1 hour before centrifugation. After first homogenization followed with 10 minutes´ incubation, samples were homogenized again and one aliquot transferred into empty tube to be centrifuged, while second aliquot was left to soak for additional 50 minutes before centrifugation (*14*, *16*). Furthermore, since weighing method and Calex device differ in buffer volume used for extractions, we found to be convenient to study the extraction yield with different sample dilutions for fCAL® Turbo method. Thirteen samples were extracted using Calex and additionally weighted and extracted 3 times with Bühlmann extraction buffer to gain 1:50, 1:100 and 1:500 diluted extracts. After complete homogenization and incubation, extracts were centrifuged as described before, followed with further dilution of 1:50 and 1:100 diluted extracts to gain desirable 1:500 dilutions.

### Calprotectin measurements

Based on study protocol (Figure 1), extracts were analysed with fluorescence enzyme immunoassay (FEIA), EliA^TM^ Calprotectin on Phadia 100 analyser (Thermo Fisher, Uppsala, Sweden) and/or PETIA, Bühlmann fCAL® Turbo on Roche Cobas c501 analyser (Roche diagnostics, Manheim, Germany). Measuring ranges for FEIA and PETIA are 15 - 3000 mg/kg (within-laboratory coefficient of variation, CV = 4.8%) and 20 - 2000 mg/kg (within-laboratory CV = 4.9%), respectively. Cut-off value for both methods is set at 50 mg/kg (negative). Values 50 - 200 mg/kg represent the “grey zone” and > 200 mg/kg active inflammation in gastrointestinal tract (*14*, *22*).

### Statistical analysis

All data sets were tested for normality using the Shapiro-Wilk test. Outliers were detected using Tukey test. Statistical tests were used as follows.

#### Within-method variability with respect to extraction method used and stool consistency

Due to non-normally distributed data, results were presented as median and interquartile range (IQR) and Wilcoxon test used to assess the agreement between different extraction procedures. Bias (B) was calculated using the following equation: B = [(FC_X_ - FC_R_) / FC_R_ ] x 100, where FC_X_ represents FC concentration (mean values of double performed extractions, where appropriate) gained in extraction devices’ extracts and FC_R_ represents FC concentration in the reference weighing method extract. Mean of calculated biases was compared with allowable bias set at 22.6%. Allowable bias was calculated using the formula 0.250 x (CVi^2^ + CVg^2^)^0.5^, where CVi represents the within-subject and CVg between-subject biological variation (*23*). Data for CVi and CVg were taken from recent study conducted by Padoan and co-authors, and for PETIA method are 32.3% and 84.3%, respectively (*24*). Intra-extraction precision was estimated from duplicate FC values of double-extracted samples and expressed as mean of calculated coefficient of variations (CV = standard deviation / mean of double-extracts).

#### Between-method variability with respect to the extraction method used and stool consistency

Due to non-normally distributed data, results were presented as median and IQR. Method comparison was done using Wilcoxon test and Bland-Altman plot. Biases between two methods were calculated using the formula B = [(FC_B_ – FC_E_) / ((FC_B_ + FC_E_)/2)] x 100, where FC_B_ represents FC concentration gained with fCAL® Turbo method and FC_E_ with EliA^TM^ Calprotectin method. Faecal calprotectin results for both methods were additionally divided into two categories: < 200 mg/kg (negative and “grey zone” values) and > 200 mg/kg (high values, inflammation likely), biases calculated according to previous formula and Wilcoxon test and Bland-Altman plot were applied. Mean of calculated biases was compared to desirable bias of 22.6%. Additionally, differences in estimated biases between extraction methods, different methods comparison and differences in CVs calculated for intra-extraction precision based on the stool consistency category were tested using non-parametric Kruskal-Wallis ANOVA and Conover *post-hoc* test due to small sample number *per* group.

#### Evaluation of extraction yield in regard to incubation time and initial sample dilution

Extraction yield was analysed using Wilcoxon test and Friedman ANOVA with Conover *post-hoc* test. Bias from reference FC concentration gained for 10 min extracts was calculated using the following equation: B = [(FC_1hour_ – FC_10min_) / FC_10min_ ] x 100, and mean of calculated biases compared to defined criteria.

Statistical analysis was performed using the MedCalc statistical software, version 14.8.1 (Ostend, Belgium). Values of P < 0.05 were considered statistically significant.

## Results

### Within-method variability with respect to extraction method used and stool consistency

Median FC values measured in extracts gained with EliA^TM^ and Bühlmann fCAL® Turbo related extractions procedures together with corresponding mean biases are summarized in Table 1. Median FC concentration in Calex extracts was significantly higher (P < 0.001) in comparison to weighing method extracts with calculated mean bias of 33.3%, which exceeded acceptable criteria. For EliA^TM^ Calprotectin method no statistically significant difference was found between different extraction procedures (P = 0.484). There were no statistically significant differences in biases between results categorized in 3 classes according to stool consistency (P = 0.300 EliA^TM^, P = 0.697 fCAL® Turbo).

**Table 1:** Variability within EliA^TM^ Calprotectin and Bühlmann fCAL Turbo method for measuring faecal calprotectin with respect to extraction method and stool consistency

				**Mean biases by BSS, %**			
**Extraction method**	**FC concentration, mg/kg**	**P^†^**	**Mean bias, %**	Hard Stool	Normal Stool	Liquid Stool	**P^‡^**
EliA^TM^ Calprotectin, N = 52*							
Weighing method	125.0 (43.0 - 423.5)	0.484	0.1	- 8.9 (N = 7)	2.5 (N = 31)	- 0.8 (N = 14)	0.300
EliA Stool Extraction Kit extraction device	116.5 (36.0 - 407.5)						
Bühlmann fCAL® Turbo, N = 85*							
Weighing method	140.0 (48.8 - 430.0)	< 0.001	33.3	34.3 (N = 14)	33.9 (N = 54)	33.3 (N = 17)	0.697
Calex® Cap “N” extraction device	167.0 (73.0 - 544.0)						
*2 samples out of 54 and 5 out of 90 were excluded as outliers. Faecal calprotectin (FC) concentration is presented as median (interquartile range).^†^Comparison of medians was performed using the Wilcoxon test. Mean bias – mean of calculated biases (Bias = (FC_extraction device_ – FC_weighing_) / FC_weighing_ x 100). BSS- Bristol stool scale classes (hard- BSS 1 and 2; normal- BSS 3, 4, 5; liquid- BSS 6, 7). ^‡^Comparison of biases was performed using the Kruskal-Wallis test. P < 0.05 was considered statistically significant. The allowable bias was set at 22.6%.							

Mean CVs calculated from double-performed extractions by extraction devices ranged from 3.5% (2.5 - 4.5) for EliA SEK to 9.7% (6.4 - 13.0) for Calex. Coefficients of variation did not differ statistically significant between 3 classes of results according to stool consistency (P = 0.366 EliA SEK; P = 0.194 Calex).

### Between-method variability with respect to the extraction method used and stool consistency

**Figure 2:**
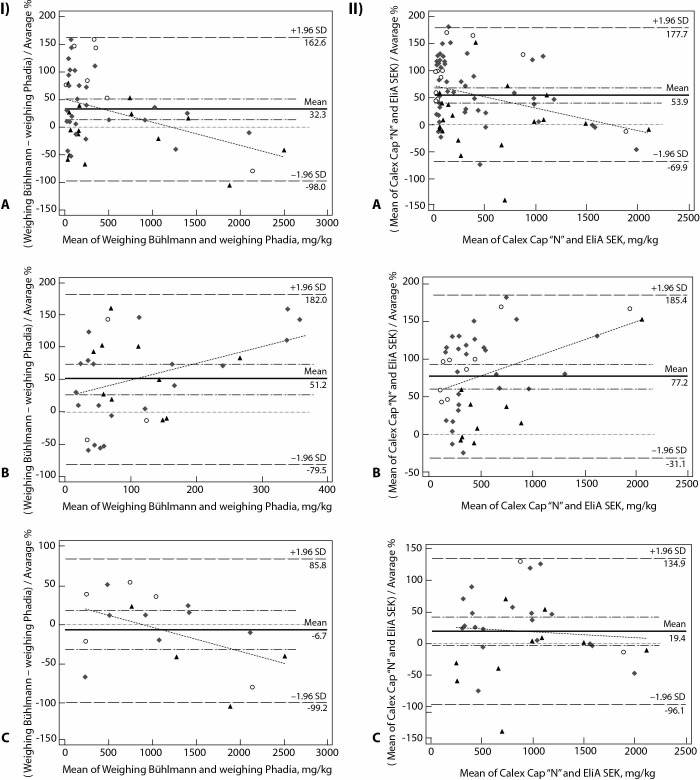
Bland-Altman plot of faecal calprotectin (FC) values between EliA™Calprotectin and Bühlmann fCAL® Turbo with regard to extraction method used. I) Comparison of weighing methods: A) comparison of all FC results (N = 52); B) comparison of FC results < 200 mg/kg (N = 35); C) comparison of FC results > 200 mg/kg (N = 17). II) Comparison of volume-based extraction devices EliA Stool Extraction Kit (EliA SEK) *vs* 6-groove Bühlmann Calex® Cap “N”: A) comparison of all FC results (N = 77); B) comparison of FC results < 200 mg/kg (N = 46); C) comparison of FC results > 200 mg/kg (N = 31). The difference in Bühlmann fCAL Turbo and EliA^TM^ Calprotectin FC concentration is plotted against the mean FC concentration of both methods. Different shapes of markers represent different stool consistency category: circle - hard (BSS 1, 2); diamonds - normal (BSS 3, 4, 5); triangle - liquid (BSS 6, 7). BSS – Bristol stool scale.

Comparison results are summarized in Table 2 and Figure 2. Faecal calprotectin results obtained with fCAL® Turbo method were significantly higher compared to EliA^TM^ method irrespective of extraction method used. Mean calculated biases of 32.3% and 53.9% for weighing methods and extraction devices comparison, respectively, exceeded allowable bias of 22.6%. When results were categorized in classes according to stool consistency, bias was acceptable only for liquid stool group where there was no statistically significant difference between FC results gained with two methods (weighing methods P = 0.980; EliA SEK *vs* Calex P = 0.541). Regression lines in Bland-Altman plots show that biases between two methods are becoming lower with higher FC concentration (Figure 2). Followingly, division of results into two categories revealed statistically significant difference between two methods in group with FC values < 200 mg/kg with fCAL® Turbo results being higher than EliA^TM^ by 51.2% (P < 0.001) for weighing methods and by 77.2% for EliA SEK *vs* Calex (P < 0.001). In the group with FC values > 200 mg/kg there was no statistically significant difference between two methods with EliA^TM^ results being higher than fCAL® Turbo by mean 6.7% (P = 0.781) for weighing method and lower by mean 19.4% for EliA SEK *vs* Calex (P = 0.064).

**Table 2:** Between-method variability for measuring faecal calprotectin with respect to the extraction method used and stool consistency

			**Hard Stool**		**Normal Stool**		**Liquid Stool**		
	**FC concentration mg/kg**	P*	**FC concentration, mg/kg**	**Mean bias,%**	**FC concentration, mg/kg**	**Mean bias,%**	**FC concentration, mg/kg**	**Mean bias,%**	**P^†^**
**Weighing method comparison, N = 52**									
EliA™ Calprotectin	104.0 (36.0 - 415.0)	0.025	84.8 (22.0 - 257.5)	87.9 (N = 8)	74.0 (26.0 - 217.5)	38.1 (N = 29)	162.0 (58.5 - 1045.5)	- 8.4 (N = 15)	0.005 (*1*)/ (*2*)/ (*3*)
Bühlmann fCAL® Turbo	160.5 (59.5 - 611.0)		491.5 (127.0 - 611.0)		124.0 (57.8 - 373.5)		152.0 (66.5 - 929.3)		
**EliA Stool Extraction Kit *vs* Calex^®^ Cap “N”, N = 77**									
EliA™ Calprotectin	91.0 (38.1 - 453.5)	< 0.001	27.0 (19.3 - 61.3)	89.2 (N = 11)	77.0 (32.3 - 390.0)	64.1 (N = 47)	277.0 (88.8 - 924.3)	8.1 (N = 19)	< 0.001 (*3*)/ (*1*) (*2*)
Bühlmann fCAL® Turbo	190.5 (76.3 - 710.9)		103.5 (41.1 - 594.1)		249.0 (74.8 - 555.9)		203.5 (95.8 - 999.1)		
FC - faecal calprotectin concentration is presented as median (interquartile range). *Wilcoxon test. Stool classes are presented according to Bristol stool scale (BSS): hard (BSS 1, 2), normal (BSS 3, 4, 5) and liquid (BSS 6, 7). ^†^Kruskal-Wallis test with Conover *post-hoc* pairwise comparison. Mean bias - mean of calculated biases (Bias = [(FC_fCAL Turbo_ – FC_EliA_) / ((FC_fCAL Turbo_+FC_EliA_)/2)] x 100). P < 0.05 was considered statistically significant. The allowable bias was set at 22.6%. (*1*)/ (*2*)/ (*3*) - respective biases differ significantly between 3 classes according to stool consistency; (*3*)/ (*1*) (*2*) - bias obtained for class 3 is significantly different from respective biases obtained for classes 1 and 2 according to stool consistency.									

### Evaluation of extraction yield depending on incubation time and initial sample dilution

Results of evaluation of extraction yield are given in Table 3. Median FC concentrations after 1-hour incubation were significantly higher than after 10-minute incubation for EliA^TM^ Calprotectin and fCAL® Turbo; P = 0.019 and P < 0.001, respectively with greater deviations seen with EliA^TM^ method. Mean biases for both methods did not exceed the allowable criteria set at 22.6%. Higher initial sample dilution with extraction buffer in weighing method (tested only for fCAL® Turbo method), lead to increased extraction yield (P < 0.001), but no significant difference was found between weighing method with 1:500 initial dilution and Calex device which originally employs 1:500 dilution.

**Table 3:** Evaluation of extraction yield depending on incubation time and initial sample dilution

**Extraction yield with different sample incubation time in extraction buffer, N = 20**			
	**FC concentration, mg/kg**	P*	**Mean bias, %**
EliA™ Calprotectin, 10 min	128.5 (39.8 - 334.0)	0.019	16.5
EliA™ Calprotectin, 1 hour	155.0 (44.0 - 378.0)		
Bühlmann fCAL^®^ Turbo, 10 min	112.0 (54.0 - 281.5)	< 0.001	6.5
Bühlmann fCAL^®^ Turbo, 1 hour	120.5 (56.0 - 319.0)		
**Extraction yield with different initial FC sample dilution, Bühlmann fCAL Turbo, N = 13**			
	**FC concentration, mg/kg**	**P^†^**	**Multiple comparison** **(Conover *post-hoc*)**
Weighing 1/50 (*1*)	361.0 (63.5 - 676.0)	< 0.001	(*1*)/ (*2*)/ (*3*) (*4*)
Weighing 1/100 (*2*)	378.0 (81.5 - 884.5)		
Weighing 1/500 (*3*)	384.0 (81.8 - 1009.3)		
Calex^®^ Cap “N” (*4*)	403.0 (94.0 - 913.0)		
FC-faecal calprotectin concentration is presented as median (interquartile range). *Wilcoxon test. Mean bias - mean of calculated biases (Bias = [(FC_1hour_ – FC_10min_) / FC_10min_ ] x 100). ^†^Friedman ANOVA with Conover *post hoc*. P < 0.05 was considered statistically significant. The allowable bias was set at 22.6%. (*1*)/ (*2*)/ (*3*) (*4*) - median FC value of 1/50 group is significantly different from median of 1/100 group and this two medians are significantly different from medians of 1/500 and Calex^®^ Cap “N” group.			

## Discussion

The results of our study have shown that FC concentrations measured with EliA^TM^ Calprotectin are significantly lower than Bühlmann fCAL® Turbo results, and the difference is dependent on extraction method used, stool consistency and FC concentration. EliA SEK device showed better precision and agreement with weighing method compared to Calex device irrespective of stool consistency. Use of the new version of Calex device (contain 6 grooves) in our study confirmed that removal of two grooves, did not reduced the previously obtained difference between FC results from 8-groove Calex and weighing method extracts, as expected (*8*, *25*, *26*).

Our results indicate that manipulation of the sample with extraction buffer influences the extraction yield. Longer incubation in buffer resulted in higher yield, irrespective of assay, while increased extraction yield obtained with higher initial sample dilution could be one of the main reasons for the significant difference between weighing method and Bühlmann´s extraction device. Namely, as mentioned previously, EliA SEK yields 1:50 diluted extracts as well as weighing method and then both extracts are further diluted on analyser to required 1:500 dilution. On contrary, Bühlmann´s extraction methods use different sample extraction protocols, Calex extracts are ready to use (1:500) compared to weighing method which yields 1:50 diluted extracts with afterward manual dilution to required 1:500 dilution. Our conclusion is in line with the finding of Tøn and co-authors who also perceived a tendency towards increased extraction yield with higher sample dilutions and longer homogenization time (*17*). Possible explanation for this influence is that buffer composition and capacity, which is the property of the manufacturers, could be responsible for different extraction yield.

Impact of the initial sample dilution and stool amount is reflected on the devices’ precisions since for EliA SEK it is minimal (grooves carry approximately 15 mg of 1:50 diluted sample), while for Bühlmann´s 6-groove Calex is somewhat higher (grooves carry approximately 8 mg of 1:500 diluted sample). In our study, performance of volume–based devices showed to be comparable to weighing method with regard to stool consistency. Results from previous studies regarding performances of extraction devices with liquid stool sample are contradictory (*11*, *18*, *20*). Oyaert and colleagues obtained an underestimation of FC concentration with EliA SEK performed extractions compared to Smart Prep device (*11*). Kristensen gained similar results for 8-groove Calex Cap® device with intra-extraction precision CV for liquid stool up to 33.3% (*7*). It’s assumed that extraction devices are unable to withhold sufficient amount of stool sample leading in increased variability of results (*7*, *8*, *16*). Conversely, another study showed excellent correlation of Calpro EasyExtract device (similar design as EliA SEK) with Smart Prep device demonstrating that devices’ grooves are able to maintain even liquid stool sample (*18*).

Comparison of EliA^TM^ Calprotectin and fCAL Turbo methods yielded significant proportional difference between methods. Higher results were obtained for fCAL® turbo method with mean bias from 32.3% for weighing methods comparison, up to 53.9% for EliA SEK *vs* Calex. Higher biases between extraction devices compared to those between weighing methods could also be explained with different initial sample dilution employed, and devices’ different design. Observed biases were lower with higher FC results so that in category > 200 mg/kg they became insignificant. Although this measuring area is of the utmost clinical importance, different categorization in the area of the lower results could have an impact on diagnosis of the mild organic disease. Oyaert compared analytical performances of 6 different assays for FC determination including Bühlmann fCAL® turbo and EliA^TM^ Calprotectin 2 (second re-standardized generation of EliA calprotectin which is not available for Phadia 100) using Smart Prep device for stool sample extraction. He found variations in FC concentration between different methods, which does not allow their interchangeable use (*9*). At the same time, EliA^TM^ Calprotectin 2, which requires 1:75 prediluted samples, showed somewhat higher results than fCAL® turbo, although not statistically significant. In conclusion, the question arises whether the same cut-off can be appropriate for different methods.

Obtained significant difference between two methods based on stool consistency is in line with our conclusion that longer incubation time (expected for hard stool samples) together with higher initial sample dilution in applied extraction methods leads to FC increased extraction yield.

One of the limitations to the study is that we did not perform the study of diagnostic accuracy. In addition, the different levels of achieved homogeneity due to the sample consistency or the admixture of some indigested particles inevitably influenced our results. On the other hand, this could be regarded as general limitation of studies with stool samples. We believe that the most important contribution of our study lies in the extraction yield evaluation. To the best of our knowledge, this is the first study that evaluated the variability of Calex Cap® “N” device regarding weighing and EliA^TM^ Calprotectin method with respective number of stool samples with different consistencies. However, extraction yield evaluation should be further supported with larger sample size of stools with different consistencies.

To conclude, within-method differences depending on the extraction method applied as well as between-method differences are highly influenced by pre-analytical FC sample management. This might prompt manufacturers to apply customized protocols regarding sample-soaking time and initial sample dilutions based on extraction buffer capacity and method requirements, which would contribute to FC standardization process.
